# Association of menopausal status with COVID-19 outcomes: a propensity score matching analysis

**DOI:** 10.1186/s13293-021-00363-6

**Published:** 2021-01-29

**Authors:** Xing-Wang Wang, Hao Hu, Zhi-Yong Xu, Gong-Kai Zhang, Qing-Hua Yu, Hui-Lan Yang, Ji-Hua Zheng

**Affiliations:** 1 Department of Dermatology, General Hospital of Southern Theater Command, No. 111 Liuhua Road, Guangzhou, 510010 People’s Republic of China; 2 Department of Radiation Therapy, General Hospital of Southern Theater Command, No. 111 Liuhua Road, Guangzhou, 510010 People’s Republic of China; 3 Department of Emergency, General Hospital of Southern Theater Command, Guangzhou, People’s Republic of China

**Keywords:** COVID-19, Menopause status, Mortality, Disease severity, Propensity score matching

## Abstract

**Background:**

Despite the growing number of studies on the coronavirus disease-19 (COVID-19), little is known about the association of menopausal status with COVID-19 outcomes.

**Materials and methods:**

In this retrospective study, we included 336 COVID-19 inpatients between February 15, 2020 and April 30, 2020 at the Taikang Tongji Hospital (Wuhan), China. Electronic medical records including patient demographics, laboratory results, and chest computed tomography (CT) images were reviewed.

**Results:**

In total, 300 patients with complete clinical outcomes were included for analysis. The mean age was 65.3 years, and most patients were women (*n* = 167, 55.7%). Over 50% of patients presented with comorbidities, with hypertension (63.5%) being the most common comorbidity. After propensity score matching, results showed that men had significantly higher odds than premenopausal women for developing severe disease type (23.7% vs. 0%, OR 17.12, 95% CI 1.00–293.60; *p* = 0.003) and bilateral lung infiltration (86.1% vs. 64.7%, OR 3.39, 95% CI 1.08–10.64; *p* = 0.04), but not for mortality (2.0% vs. 0%, OR 0.88, 95% CI 0.04–19.12, *p* = 1.00). However, non-significant difference was observed among men and postmenopausal women in the percentage of severe disease type (32.7% vs. 41.7%, OR 0.68, 95% CI 0.37–1.24, *p* = 0.21), bilateral lung infiltration (86.1% vs. 91.7%, OR 0.56, 95% CI 0.22–1.47, *p* = 0.24), and mortality (2.0% vs. 6.0%, OR 0.32, 95% CI 0.06–1.69, *p* = 0.25).

**Conclusions:**

Men had higher disease severity than premenopausal women, while the differences disappeared between postmenopausal women and men. These findings support aggressive treatment for the poor prognosis of postmenopausal women in clinical practice.

**Supplementary Information:**

The online version contains supplementary material available at 10.1186/s13293-021-00363-6.

## Introduction

Coronavirus disease 2019 (COVID-19), caused by severe acute respiratory syndrome coronavirus 2 (SARS-CoV-2), has led to severe illness and death all over the world [1–4]. As of September 13, 2020, there were over 28 million confirmed cases and over 917,000 related deaths reported worldwide [5]. This pandemic poses a serious challenge to the global health system and world economy.

With the increasing studies on COVID-19, the association of sex differences (i.e., between men and women) with the clinical outcomes started to be reported. Sex differences in the innate and adaptive immune systems lead to differences in the susceptibility and response to SARS-CoV-2, as well as incidence and disease severity [6]. However, the differences in COVID-19 outcomes between men and women are inconsistent [1, 4, 7, 8], emphasizing the need to understand the sex-related COVID-19 outcomes.

Biological sex plays a multidimensional role in COVID-19 outcomes, including complex interactions based on sex chromosome complement, reproductive tissues, and the concentration of sex steroid hormones (estrogen, progesterone, and testosterone) [9]. Despite several studies investigating common susceptibility factors (older age, sex, comorbidities) to virus infection [10, 11], little is known about the association of menopause status (i.e., premenopause and postmenopause) with clinical outcomes of hospitalized patients with COVID-19. As previously reported [12], postmenopausal women showed a significant change in the concentration of plasma sex hormones (e.g., estrogen and progesterone depletion) compared with premenopausal women. In addition, estrogen and/or progesterone may regulate the innate and adaptive immune response [13, 14]. However, whether premenopausal and postmenopausal women exhibited different COVID-19 outcomes remains unclear.

In the present study, we conducted a retrospective analysis, using the propensity score matching method to minimize selection bias and control potential confounding factors, to separately compare COVID-19 outcomes of hospitalized premenopausal women, postmenopausal women, and men.

## Materials and methods

### Study design and patient selection

As shown in Supplementary Figure 1, this retrospective study included 336 consecutive adult patients admitted with COVID-19—confirmed by reverse transcription polymerase chain reaction (RT-PCR) for SARS-CoV-2 infection—between February 15, 2020 and April 30, 2020 at Taikang Tongji Hospital (Wuhan, China) that was a temporarily designated hospital for treating patients with COVID-19 [15]. After initial screening, 36 of the 336 patients were excluded due to missing data. Thus, we estimated the sex difference outcomes in the remaining 300 patients. This study was approved by the Ethics Committee of General Hospital of Southern Theater Command of PLA. Because of the retrospective design and anonymous data used in the study, written informed consent was not obtained.

### Propensity score matching

As previously reported [16], propensity score matching can balance the treatment and control groups based on baseline covariates to minimize the bias factors and control potential confounding factors. Therefore, we performed propensity score matching at a ratio of 1:1 nearest-neighbor matching algorithm to adjust for age, body mass index (BMI), comorbidities (hypertension, diabetes, cerebrovascular disease, chronic obstructive pulmonary disease, coronary heart disease, and cancer), treatment (corticosteroids, arbidol, and interferon), and laboratory results (lactic dehydrogenase, lymphocyte, and C-reactive protein). Finally, a well-balanced cohort of 101 paired patients (men and women) was analyzed (Table 1).

**Table 1:** Baseline characteristics before and after matching cohorts

Characteristics	No. (%) before PSM				No. (%) after PSM			
	Total	Men (***N*** = 133)	Women (***N*** = 167)	***p*** value	Total	Men (***N*** = 101)	Women (***N*** = 101)	***p*** value
Age (mean± SD), years	65.3 ± 14.6	65.6 ± 14.6	65.0 ± 14.7	0.74	65.3 ± 14.8	65.2 ± 14.9	65.4 ± 14.7	0.92
Age ≥ 65	148 (49.3)	78 (58.6)	70 (41.9)	0.004*	83 (41.1)	41 (40.6)	42 (41.6)	0.89
Age < 65	152 (50.7)	55 (41.4)	97 (58.1)		119 (58.9)	60 (59.4)	59 (58.4)	
BMI								
Under and normal weight	220 (73.3)	93 (69.9)	127 (76.0)	0.47	146 (72.2)	71 (70.3)	75 (74.2)	0.3
Overweight	69 (23.0)	35 (26.3)	34 (20.4)		49 (24.3)	28 (27.7)	21 (20.8)	
Obese	11 (3.7)	5 (3.8)	6 (3.6)		7 (3.5)	2 (2.0)	5 (5.0)	
Comorbidities								
Hypertension	196 (65.3)	94 (70.7)	102 (61.1)	0.08	130 (64.4)	66 (65.3)	64 (63.4)	0.77
Diabetes	57 (19.0)	26 (19.5)	31 (18.6)	0.83	37 (18.3)	21 (3.6)	16 (3.6)	0.36
Cancer	8 (26.7)	5 (3.8)	3 (1.8)	0.29	3 (1.5)	2 (20.8)	1 (1.0)	0.56
Coronary heart disease	57 (19.0)	24 (18.0)	33 (19.8)	0.71	39 (19.3)	18 (17.8)	21 (20.8)	0.59
COPD	39 (13.0)	24 (61.5)	15 (38.5)	0.02*	22 (10.9)	12 (11.9)	10 (10.0)	0.65
Cerebrovascular disease	30 (10.0)	14 (46.7)	16 (53.3)	0.79	25 (12.4)	11 (10.0)	14 (13.9)	0.52
Treated with corticosteroids								
Yes	19 (6.3)	13 (9.8)	6 (3.6)	0.03*	9 (4.5)	3 (3.0)	6 (5.9)	0.31
No	281 (93.7)	120 (90.2)	161 (96.4)		193 (95.5)	98 (97.0)	95(94.1)	
Treated with interferon								
Yes	35 (11.7)	16 (12.0)	19 (11.4)	0.86	176 (87.1)	88 (87.1)	88 (87.1)	1
No	265 (88.3)	117 (88.0)	148 (88.6)		26 (12.9)	13 (12.9)	13 (12.9)	
Treated with arbidol								
Yes	153 (51.0)	77 (57.9)	76 (45.5)	0.03*	101 (50.0)	49 (48.5)	52 (51.5)	0.67
No	147 (49.0)	56 (42.1)	91 (54.5)		101 (50.0)	52 (51.5)	49 (48.5)	
Lactic dehydrogenase								
Abnormal	38 (12.7)	20 (15.0)	18 (10.8)	0.27	180 (89.1)	91 (90.1)	89 (88.1)	0.65
Normal	262 (87.3)	113 (85.0)	149 (89.2)		22 (10.9)	10 (9.9)	12 (11.9)	
Lymphocyte								
Abnormal	207 (69.0)	81 (60.9)	126 (75.4)	0.007*	61 (30.2)	29 (28.7)	32 (31.7)	0.65
Normal	93 (31.0)	52 (39.1)	41 (24.6)		141 (69.8)	72 (71.3)	69 (68.3)	
C-reaction protein								
Abnormal	70 (23.3)	41 (30.8)	29 (17.4)	0.006*	161 (79.7)	80 (79.2)	81 (80.2)	0.86
Normal	230 (76.7)	92 (69.2)	138 (82.6)		41 (20.3)	21 (20.8)	20 (19.8)	

Abbreviations: *PSM* propensity score matching, *SD* standard deviation, *COPD* chronic obstructive pulmonary disease

*Significant at *p* < 0.05

### Data collection

We reviewed the medical records and extracted the following data: age, sex (including menopause status for women), body mass index (BMI), comorbidities (hypertension, diabetes, cerebrovascular disease, chronic obstructive pulmonary disease, coronary heart disease, and cancer), treatment (corticosteroids, arbidol, and interferon), laboratory results (lactic dehydrogenase, lymphocyte, and C-reactive protein), and chest computed tomography (CT) images. According to the criteria of the Working Group on Obesity in China [17], we categorized BMI (calculated as weight in kilograms divided by height in meters squared) into three groups: underweight and normal weight, BMI < 24 kg/m^2^; overweight, 24 ≤ BMI < 28 kg/m^2^; and obese, BMI ≥ 28 kg/m^2^.

### Definition and outcomes

Menopause is defined retrospectively as the cessation of spontaneous menstruation for 12 months [18]. The primary outcomes were disease severity, mortality, and chest CT imaging features. As previously reported [1], COVID-19 disease severity was divided into severe or non-severe. Severe disease should meet any of the following criteria: (a) respiratory rate ≥ 30 breaths per min, (b) oxygen saturation ≤ 93% in a resting state, (c) ratio of arterial oxygen partial pressure and oxygen concentration ≤ 300 mmHg, and (d) progression of more than half of lesions in lung imaging within 1–2 days. Chest CT imaging features were classified as unilateral lung infiltration or bilateral lung infiltration based on a CT diagnosis report provided by experienced thoracic radiologists in the electronic medical records.

### Statistical analysis and subgroup analysis

To investigate the impact of menopausal status on COVID-19 outcomes, we compared clinical outcomes in three groups of well-balanced and case-matched COVID-19 patients: men, premenopausal women, and postmenopausal women. All analyses were conducted using SPSS (version 22.0, IBM SPSS Inc., Chicago, IL, USA) and GraphPad Prism 6.07 (GraphPad Software, Inc., La Jolla, CA, USA). Continuous and categorical variables were presented as mean (standard deviation) and number (%), respectively. Continuous variables were compared using unpaired Student’s *t* test. Categorical variables were compared using chi-square test or Fisher’s exact test, if appropriate. All statistical significance was two-sided and set at *p* value less than 0.05.

## Results

### Study cohort characteristics before and after score matching analysis

As of April 30, 2020, we collected the medical records of 336 patients hospitalized with COVID-19. Of these patients, 36 patients were excluded due to missing data: two patients without complete outcomes, 11 without data of blood routine examination, 15 without data of lactic dehydrogenase, and 8 without height and/or weight data. Thus, 300 patients (89.3%, 300/336) with complete clinical information were enrolled in our study (Supplementary Figure 1). The basic characteristics of the study population before and after score matching analysis are presented in Table 1. Before score matching analysis, the mean age was 65.3 ± 14.6 years, ranging from 14 to 95 years, and most patients were female (*n* = 167, 55.7%). Comorbidities were present in in over 50% of patients, with hypertension (63.5%) being the most common comorbidity, followed by diabetes (19.0%) and coronary heart disease (19.0%). Several baseline characteristics differed significantly between the men and women groups (Table 1), such as the percentages of patients ≥ 65 years (*p* = 0.004) and patients treated with corticosteroids (*p* = 0.02) and arbidol (*p* = 0.03). After propensity score matching analysis, 202 patients were identified and the baseline characteristics of the patients were well balanced between groups (Table 1). A total of 68 patients developed severe disease type (33.7%), of whom 51.5% (35/68) were women. CT chest imaging revealed bilateral lung infiltration in most patients (86.6%, 175/202). There were 7 in-hospital deaths (3.5%), of whom 5 (71.4%, 5/7) were women.

### Clinical outcomes between men and women

**Fig. 1:**
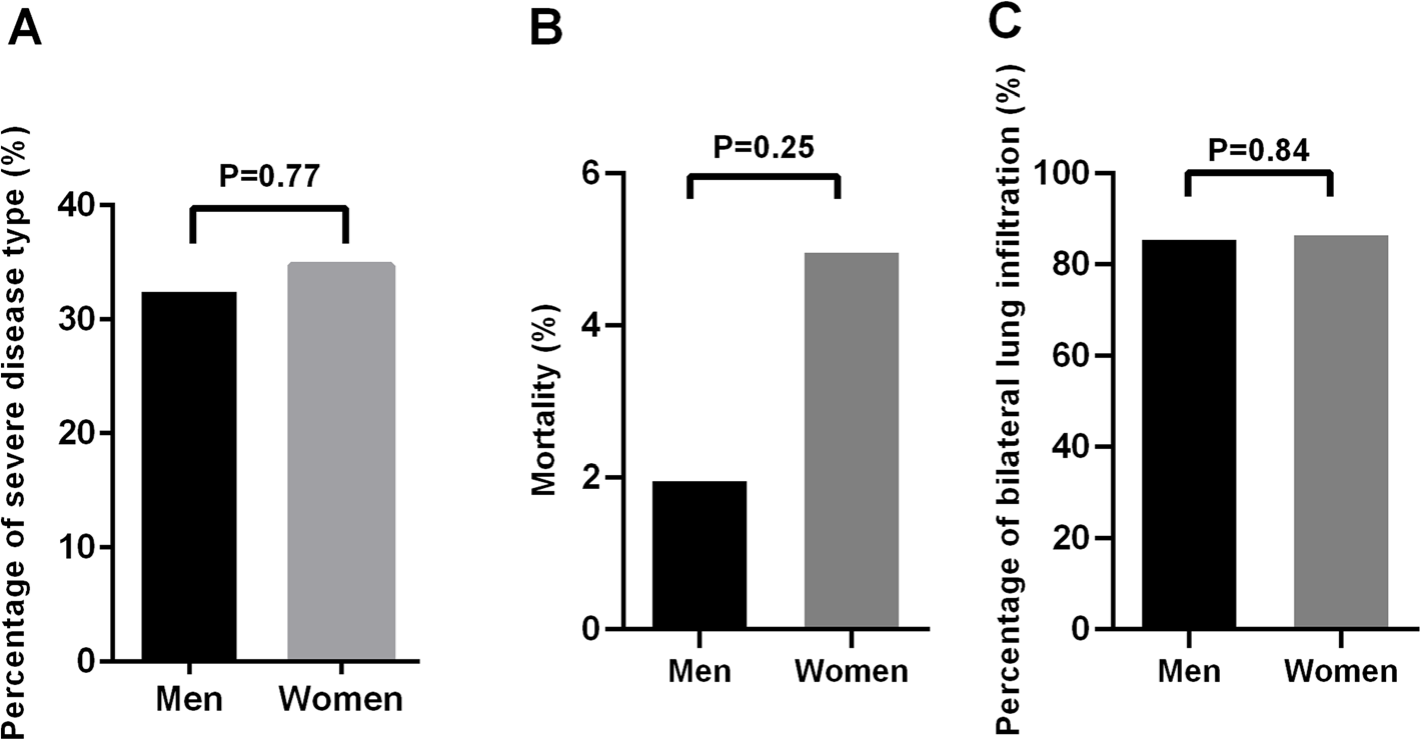
The percentage of severity type (**a**), mortality (**b**), and bilateral lung infiltration (**c**) between men and women

As shown in Fig. 1, the percentage of severe disease type (32.7% vs. 34.7%, odds ratio [OR] 0.92, 95% CI 0.51–1.64, *p* = 0.77; Fig. 1a) and bilateral lung infiltration (86.1% vs. 87.1%, OR 0.92, 95% CI 0.41–2.07, *p* = 0.84; Fig. 1c) was similar between men and women. Notably, although men showed a lower mortality rate than women, this difference was not statistically significant (2.0% vs. 5.0%, OR 0.39, 95% CI 0.07–2.05, *p* = 0.44; Fig. 1b).

### Association of menopausal status with COVID-19 outcomes

**Fig. 2:**
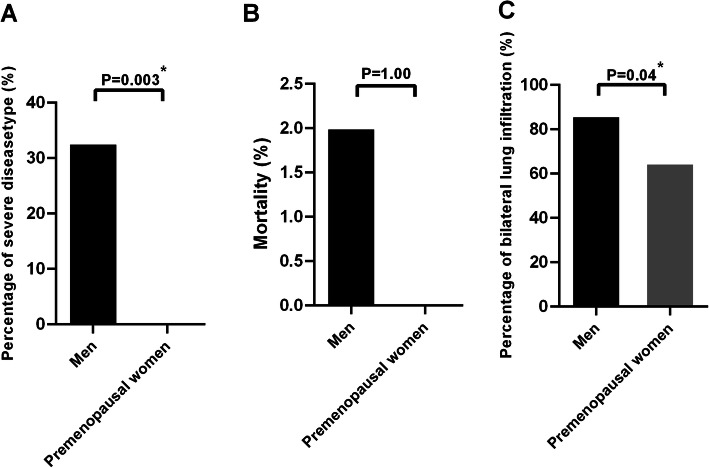
The percentage of severity type (**a**), mortality (**b**), and bilateral lung infiltration (**c**) between men and premenopausal women. *Significant at *p* < 0.05

**Fig. 3:**
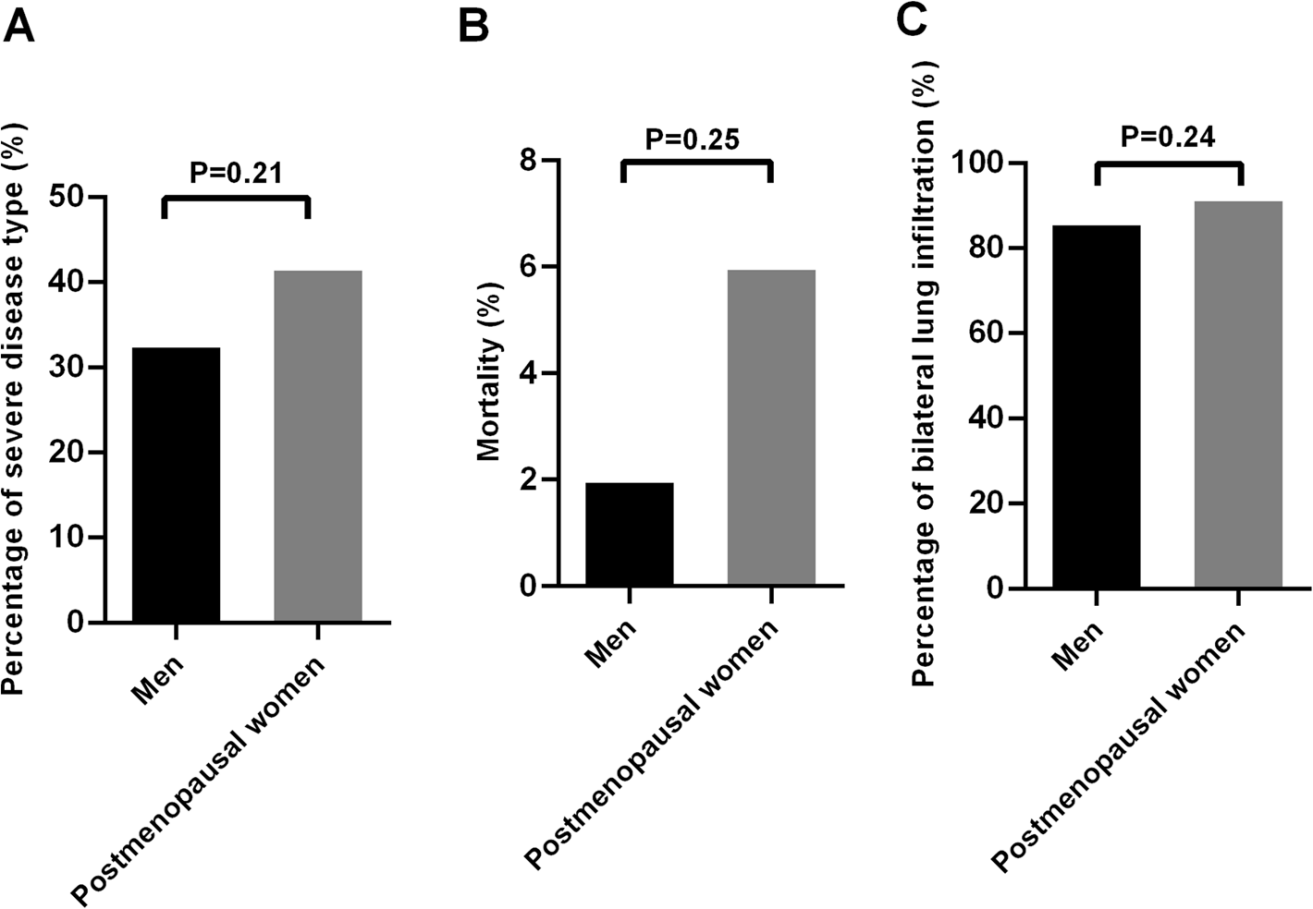
The percentage of severity type (**a**), mortality (**b**), and bilateral lung infiltration (**c**) between men and postmenopausal women

The women group was divided into two subgroups based on menopausal status: the premenopausal group and the postmenopausal group. Compared with premenopausal women, men displayed a significantly higher percentage of severe disease type (23.7% vs. 0.0%, OR 17.12, 95% CI 1.00–293.60, *p* = 0.003; Fig. 2a) and bilateral lung infiltration (86.1% vs. 64.7%, OR 3.39, 95% CI 1.08–10.64, *p* = 0.04; Fig. 2c). However, no significant difference in mortality was observed between the two groups (2.0% vs. 0%, OR 0.88, 95% CI 0.04–19.12, *p* = 1.00; Fig. 2b). In contrast, there was no difference in the percentage of severe disease type (32.7% vs. 41.7%, OR 0.68, 95% CI 0.37–1.24, *p* = 0.21; Fig. 3a) and bilateral lung infiltration (86.1% vs. 91.7%, OR 0.56, 95% CI 0.22–1.47, *p* = 0.24; Fig. 3c) between men and postmenopausal women. However, mortality still did not differ between the two groups (2.0% vs. 6.0%, OR 0.32, 95% CI 0.06–1.69, *p* = 0.25; Fig. 3b).

## Discussion

Little is known about the relationship between menopausal status and COVID-19 outcomes. To our knowledge, this is the first study comparing COVID-19 outcomes of premenopausal women and postmenopausal women with men for in-hospitalized patients based on a well-conducted propensity score matching analysis. In this study, we observed that men were significantly more likely to experience severe disease compared to premenopausal women; however, the odds of experiencing mortality was not significantly different between the two groups. Notably, the odds of experiencing severe disease and mortality were not significantly different between men and postmenopausal women. This data suggests that menopausal status bias exists in patients with COVID-19. Considering the serious health consequences and tremendous economic impacts caused by COVID-19, our findings may be useful for guiding clinicians to deploy aggressive treatment against the poor prognosis of postmenopausal women in clinical practice.

A recent retrospective study [7] reported that men were significantly associated with a higher intensive care unit (ICU) admission rate compared with women in Metropolitan Detroit. However, in this study the patients in the ICU were more likely to present with severe COVID-19 and be of older age (age > 60 years) compared with patients in the general wards. In a number of studies, the poor outcomes of older patients have been associated with reduced immune system status [19–22]. Additionally, the high proportion of cases and hospitalizations observed in women might negatively affect family structure and increase health risks and resulted in a worse prognostic profile. Jin et al. [8] also reported a high risk of severe outcomes among men with COVID-19 in China. However, this study included only a case series of 43 patients for severity analysis due to the unavailability of detailed patient information in the public data set. In addition, this smaller study for mortality analysis analyzed a heterogeneous data set. Previously published meta-analyses [23, 24] demonstrated that men had a higher prevalence of COVID-19, a higher risk of developing severe disease, and higher mortality than women. However, the clinical characteristics between the groups were unbalanced, which may have biased the results. Omar et al. [25] included 88 adult patients in Eastern Sudan and indicated that there was no significant difference in the death rate between men and women. However, this analysis was based on a small number of heterogeneous patients. Moreover, this study did not adjust for potential confounding factors such as age, BMI, comorbidities, or treatment, which were well balanced in our study by using propensity score matching analysis. Collectively, these studies may be biased in a number of ways. Firstly, they comprise relatively small sample sizes, which may result in an elevated false discovery rate or even false-positive results [26]. Secondly, the clinical characteristics between the groups were unadjusted, which was easily biased. Besides, clinical outcomes based on menopausal status were not examined in their studies. An additional limitation shared by the previous studies is that information on patient BMI and treatment medication, both known prognostic factors for COVID-19 outcomes, was not available. By contrast, in our study, BMI and treatment medication were well balanced between male and female patients. In our study, which is based on a relatively large sample size, accurate baseline and complete clinical outcome data, and propensity score matching analysis of patients’ clinical characteristics, we demonstrated that men had a significantly higher risk of developing severe COVID-19 disease than premenopausal women, but not postmenopausal women. Our results may be explained in part by the changing biochemistry due to menopause. First, loss of ovarian function at menopause and the resulting change in the concentration of sex hormones may contribute to the increased risk of COVID-19. For example, premenopausal women have higher levels of estrogen than postmenopausal women of the same age [12]. Given that estrogen plays a crucial role in protecting female mice from SARS-CoV infection and that ovariectomy or estrogen receptor blockage increases the susceptibility to infection and mortality [27], our results may be explained in part by the protective effect of estrogen against COVID-19 in premenopausal women. On the one hand, cytokine storm syndrome—an aberrant immune response to SARS-CoV-2—is one of the main reasons for the morbidity and mortality in COVID-19 [28]. In addition to its immunomodulatory effects, estrogen modulates the expression of Th1 and Th2 cytokines, deactivates excessive inflammatory processes, and restores homeostatic conditions, thus potentially inhibiting cytokine storm syndrome from occurring in women [13, 29]. On the other hand, in vitro data suggest that estrogen might exert direct antiviral activity on SARS-CoV-2 by downregulating the expression of angiotensin-converting enzyme 2 (ACE2) mRNA in bronchial epithelial cells, which has been proven to be the major receptor responsible for mediating virus entry into cells [30]. In support of this, the data from SARS-CoV-2 indicate that the use of estrogen therapy could be effective in the fight against COVID-19 [27, 31], further emphasizing the necessity of further research in patients treated with these agents. However, whether the protective effects of estrogen on COVID-19 outcomes are dose-dependent is unclear due to the unavailability of the concentration of sex hormones in the retrospective study. Second, Honour et al. reported that postmenopausal women have higher concentrations of cytokines, such as tumor necrosis factor-alpha (TNF-α), interleukin (IL)-6, and C-reactive protein, compared with premenopausal women [12]. Zhu et al. indicated that high levels of IL-6 and C-reactive protein were independent risk factors of COVID-19 severity [32]. Akbari et al. also demonstrated that the group of patients with severe COVID-19 had a significant increase in the TNF-α, IL-6, and C-reactive protein compared to the non-severe group, suggesting that these cytokines were closely associated with COVID-19 severity [33]. Hence, an increase in the concentrations of these cytokines in postmenopausal women may contribute to our findings, further emphasizing a need for increased awareness and careful monitoring of postmenopausal women. Notably, our analysis found that the mortality did not differ between the groups of men and premenopausal women, partly due to the low mortality rate observed in the limited number of premenopausal patients analyzed. However, these results should be interpreted cautiously due to the wide confidence intervals for the calculated ORs in those patients.

The present study may suffer from several limitations. First, because of the rapid emergency of the COVID-19 outbreak, our study was a retrospective analysis and not all laboratory tests were performed in all patients. Although clinical characteristics and laboratory tests were generally balanced between the men and women groups, several potentially confounding factors may not have been included and their role might be underestimated in our study. Second, the concentrations of sex hormones were unavailable due to the retrospective study design. As more data becomes available, further investigation will be required to confirm our findings. Third, the results of several clinical characteristics in our study may differ from studies in western cultures, possibly restricting the generalizability of the results. For example, previous studies mentioned that obesity (BMI > 30) was an important factor for COVID-19 outcomes; there were few individuals who were obese in the present study, compared to a significantly higher number in western cultures. Finally, the separate long-term effects of COVID-19 remain unclear and need to be evaluated further.

## Perspective and significance

Menopausal status can influence COVID-19 outcomes. Premenopausal women display lower disease severity than men, while postmenopausal women do not. Physicians should take into consideration whether women are pre- or postmenopausal when they present with COVID-19 infections, and be most aggressive in their treatment of postmenopausal women similar to their treatment of men. Further mechanistic insights are needed to determine whether menopausal status has a specific impact on the immune system response to SARS-CoV-2.

## Supplementary Information

**Additional file 1.** Supplementary figure

## Data Availability

The raw data supporting the conclusions of this article will be made available by the authors, without undue reservation.
