# Supplementary material for: Association of menopausal status with COVID-19 outcomes: a propensity score matching analysis
Source: Biol Sex Differ. 2021 Jan 29;12:16. doi: 10.1186/s13293-021-00363-6 (PMC7844785; doi:10.1186/s13293-021-00363-6)
Supplement: Supplementary file 1 — Additional file 1. Supplementary figure [file 13293_2021_363_MOESM1_ESM.docx]

*Supplementary Material*

Supplementary Figures

*
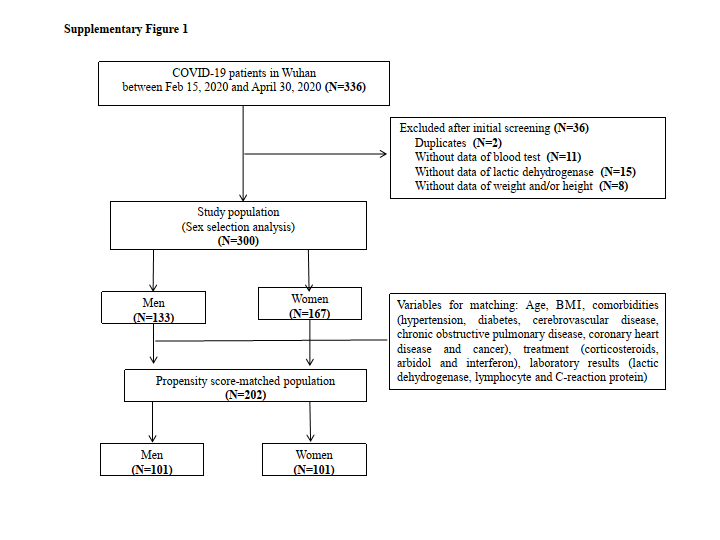
***Supplementary Figure 1. Flow diagram of the study.** COVID-19, Coronavirus Disease 2019; BMI, body mass index.
